# Shifting and reducing breathing disturbance in patients with very severe obstructive sleep apnea by modified Z-palatoplasty with one-layer closure in one-stage multilevel surgery

**DOI:** 10.1038/s41598-021-88074-1

**Published:** 2021-04-19

**Authors:** Ethan I. Huang, Yu-Ching Lin, Shu-Yi Huang, Chin-Kuo Lin, Chieh-Mo Lin

**Affiliations:** 1 Department of Otolaryngology, Chang Gung Memorial Hospital, 6 W. Sec. Jiapu Rd., Puzi, Chiayi 61363 Taiwan; 2 School of Medicine, Chang Gung University, Taoyuan, Taiwan; 3 Sleep Center of Chang Gung Memorial Hospital, Chiayi, Taiwan; 4 Division of Pulmonary and Critical Care Medicine, Chang Gung Memorial Hospital, Chiayi, Taiwan; 5 Department of Respiratory Care, Chang Gung University of Science and Technology, Chiayi, Taiwan; 6 Department of Nursing, Chang Gung University of Science and Technology, Chiayi, Taiwan; 7 Graduate Institute of Clinical Medical Sciences, College of Medicine, Chang Gung University, Taoyuan, Taiwan

**Keywords:** Surgery, Outcomes research

## Abstract

Very severe obstructive sleep apnea (OSA) with apnea–hypopnea index (AHI) ≥ 60 events/h differs in several areas from OSA with other severities, including having a low-level daytime partial pressure of oxygen and residual on-CPAP (continuous positive airway pressure) AHIs greater than 20/h. Patients with very severe OSA show narrow retroglossal space and confined framework, which is difficult to be enlarged via conventional Uvulopalatopharyngoplasty (UPPP) surgery, resulting in poor response to non-framework surgeries. Our latest report showed efficacy and efficiency for subjects undergoing modified Z-palatoplasty (ZPP) with one-layer closure in a one-stage multilevel surgery. It is unclear whether and how this procedure could help patients with very severe OSA characterized with confined framework. From Mar. 2015 to May 2018, we enrolled 12 patients with very severe OSA receiving one-stage multi-level surgery with modified ZPP with one-layer closure, CO2 laser partial tongue-base glossectomy, and bilateral septomeatoplasty. Our results show that the surgery reduced AHI from 73.8 ± 10.7 to 30.8 ± 23.2 events/h and achieved a mean AHI reduction of 58.3% (*p* < 0.001 against 0 reduction or no surgery). The surgery shifted components of the breathing disturbances. It reduced more apnea than hypopnea and might convert some apnea to hypopnea.

## Introduction

Very severe OSA is a distinct subgroup and shows differences in several areas comparing OSA with other severities. In the literature, it refers to OSA with a high AHI or respiratory disturbance index (RDI), ranging from higher than 40^1,2^, 50^3–7^, 60^8–12^, 70^13^, or 100 events/h^14^. Unlike the typical exhibition of hypoxia resulting from repeated cessations of breathing during sleep (see^15^ for a review), patients with very severe OSA may have a low level (as 77 mmHg) of diurnal partial pressure of oxygen while they are not in sleep^16^. They may have residual on-CPAP AHIs greater than 20 events/h^17^. Comparing with those with less severity, they have less positional OSA^18^, minimal inspiratory movement of the lateral pharyngeal walls and less maximum cross-sectional area^6^, higher insulin resistance^19^, a high prevalence of hypertension^16^, and about 3 times the episodes of heart block comparing with an unselected group of patients with OSA^20^.

They may comply more with regular use of the CPAP device than those with less severe OSA^21^. However, some refuse using CPAP or cannot benefit from the device. Researchers advocated alternative surgical procedures such as direct skeletal surgery^22^, bariatric surgery^23^, and tracheostomy in patients meeting certain criteria^1,24^. Typically, they have narrow retroglossal space and confined framework^22^, which is difficult to be enlarged via conventional UPPP surgery. These patients may have worse or no response to non-framework surgeries (e.g.,^9,25^).

However, the similar confined framework is commonly seen in some patients with only mild OSA. It hints an opportunity for a non-framework surgery. As a non-framework surgery, modified ZPP with one-layer closure in a one-stage multilevel surgery^26^ has revealed efficacy and efficiency as that proposed by Friedman and colleague since 2004^27–29^. It is unclear whether and how this technique could help patients with very severe OSA. Reducing OSA severity may help in its consequences, e.g., hypertension^30^, cognitive deficits^31,32^, and cardiovascular disorders^33–35^. Reducing desaturation might improve coexisting medical conditions. It is unknown how this surgery may improve the severity and desaturation. Smaller body mass index (BMI) and preoperative AHI were reported good predictors for the surgical outcome^36^. Better surgical response has been described in patients with smaller BMI (< 30) or smaller AHI (< 60)^36^. It is unclear whether they stay good predictors in the category of very severe OSA. Here in this study, we analyzed the component shifts and tested the statistical significance of pre- and post-operative sleep parameters for very severe OSA patients whose AHIs are higher or equal to 60 events/h. We also tested the effectiveness of these two predictors.

## Materials and methods

From Mar. 2015 to May 2018, we enrolled subjects with very severe OSA who met these criteria to the study:

- Age ≥ 20 years
- Unsuccessful or refusal of CPAP
- AHI ≥ 60 events/h
- Received a one-stage multi-level sleep surgery with the modified ZPP performed with one-layer closure, CO2 laser partial tongue-base glossectomy, and bilateral septomeatoplasty
- Available preoperative and postoperative polysomnography (PSG) for AHI measurement

We performed preoperative endoscopic evaluation to figure the anatomical stage^37^. Patients were not selected for surgery by any other criteria unlisted above. Epworth Sleepiness Scale (ESS) that comprises 8 4-point scale (0–3) inquiries was used to measure daytime sleepiness, with the total score ranging from 0 to 24. The higher the ESS score, the more that individual’s daytime sleepiness in everyday life. Modified ZPP with one-layer closure was carried out as illustrated in our earlier report^26^. Open tongue-base resection was completed with transoral (CO2) laser microsurgery for hypopharyngeal obstruction according to the preoperative endoscopic assessment. After the surgery, we cared all patients for in general ward areas with an oximeter monitor. Intravenous Dynastat twice daily was prescribed for 1–3 days. No intravenous or oral narcotics were given to prevent respiratory depression.

We used the percentage of reduction in mean AHI as the primary measure of surgical efficacy^38^ to compare with the results across most studies in the literature. It measures the mean change in AHI compared to the mean AHI before surgery. Mean and standard deviation summarized the pre- and post-operative AHI. We performed a paired t-test to examine the change in AHI against no change after the surgery. We examined other associated sleep parameters, including obstructive apnea index (OAI), minimum oxyhemoglobin saturation of pulse oximetry (SpO2), mean SpO2, desaturation index, and mean desaturation with a paired t-test. Postoperative care and complications were also reported, including suture dehiscence and bleeding. A p-value smaller than 0.05 was deemed significant.

We calculated the correlation coefficient between each of preoperative BMI and AHI vs. AHI reduction to test these two predictors. To clarify the effect of BMI change on AHI reduction, we computed individual BMI change (postoperative BMI–preoperative BMI in PSG records) then calculated the correlation coefficient between individual BMI change and AHI reduction. The statistical significance was tested as α = 0.05.

The statistical examinations were performed in Matlab 9.4.0.813654 (MathWorks, Natick, Massachusetts, U.S.A.).

### Ethical approval

The Institutional Review Board (IRB) of Chang Gung Medical Foundation, Taiwan approved the study methods and protocols (IRB number: 201800948B0). We performed the study in accordance with Good Clinical Practice and the applicable laws and regulations. As a retrospective cohort study, the IRB approved the waiver of the participants' consent.

## Results

Ten male and 2 female patients with very severe OSA aged between 25 and 59 years were enrolled to this work. The mean BMI was 28.1 with a standard deviation of 3.3 kg/m^2^. All patients received the 3 procedures in the multilevel sleep surgery listed above. Two and 1 patients underwent routine endoscopic sinosurgery and adenoidectomy, respectively. A PSG followed about 5 months (159 ± 59 days, mean with one standard deviation) after the surgery. Table 1 detailed the individual pre- and post-operative AHI, Friedman anatomic stage, and the surgeries performed.

**Table 1:** Enrolled subjects ordered by their visit times.

Case	Age and sex	Preop AHI	Postop AHI	Preop ESS	Postop ESS	Tonsil size	Friedman tongue position	Friedman stage	Preop body mass index	Postop body mass index	Surgeries
1	25M	79.4	29.1	17	16	3	2	1	30	32.2	abce
2	34M	66	0.8	7	8	4	3	2	28.6	29.6	abcd
3	49M	69.8	32.7	12	12	2	3	3	25.8	25.1	abc
4	40M	65.2	12.5	12	5	1	3	3	24.6	24	abc
5	50M	86.5	22	11	11	1	3	3	27.4	25.7	abc
6	31M	85.3	68.6	3	7	2	4	3	32	29.4	abc
7	58M	62.6	77.6	8	2	1	4	3	25.7	23.8	abcd
8	51M	63.1	6.4	11	4	1	4	3	21.8	23.2	abc
9	56F	92.7	13.9	18	12	1	4	3	31.5	30.3	abc
10	59M	60.8	31.5	12	14	1	2	2	27.2	24.3	abc
11	38 M	77.9	39.2	2	1	1	4	3	32.3	31.2	abc
12	58F	76.7	35.2	3	2	1	4	3	30.1	28	abc

Preop: preoperative. Postop: postoperative. Surgeries: surgical procedures in the multilevel surgery. a: modified ZPP with one-layer closure, b: open CO2-laser tongue-base resection, c: bilateral septomeatoplasty, d: endoscopic sinosurgery, e: adenoidectomy.

**Figure 1:**
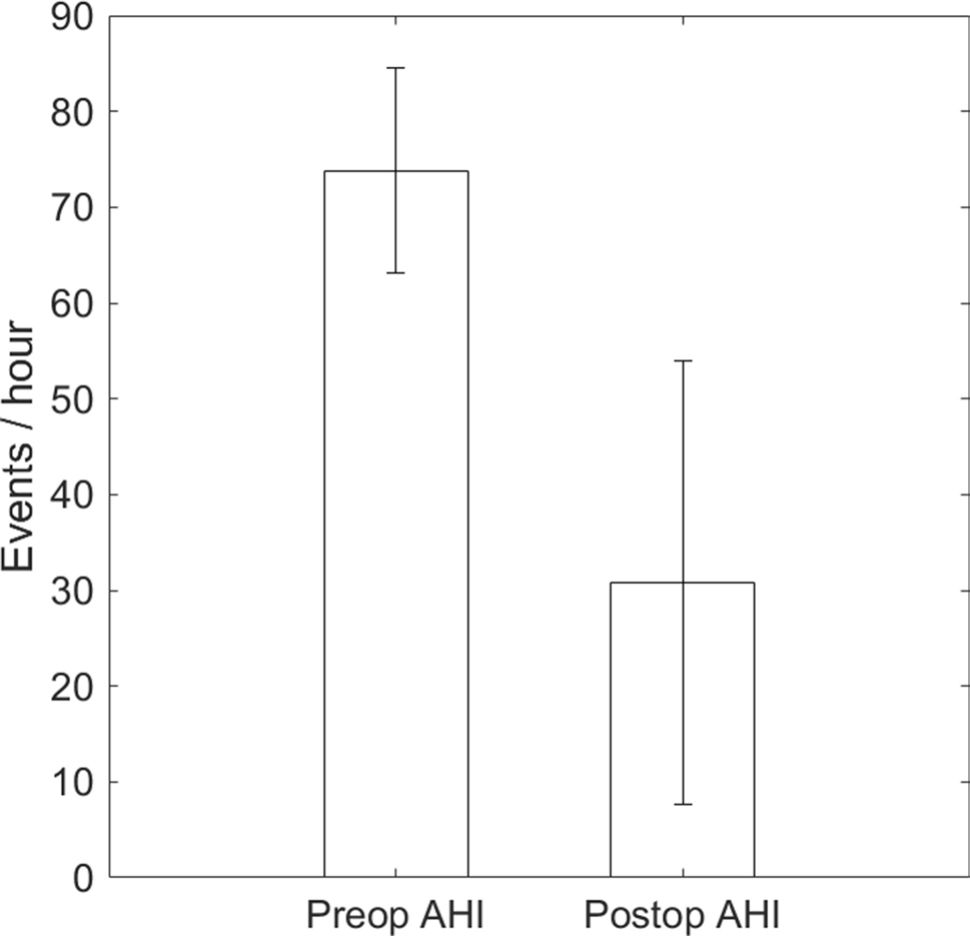
Mean and standard deviation of apnea–hypopnea index (AHI) reduced from 73.8 ± 10.7 to 30.8 ± 23.2 events/h after the surgery.

**Figure 2:**
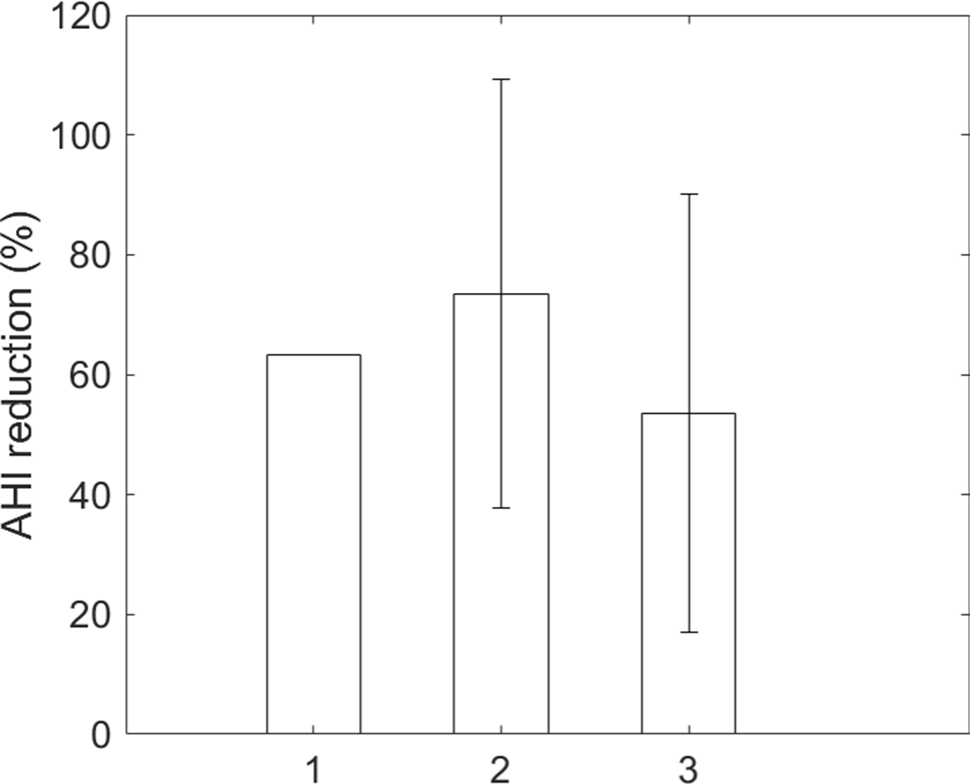
Reduction of apnea–hypopnea index (AHI) in different Friedman anatomical stage.

**Figure 3:**
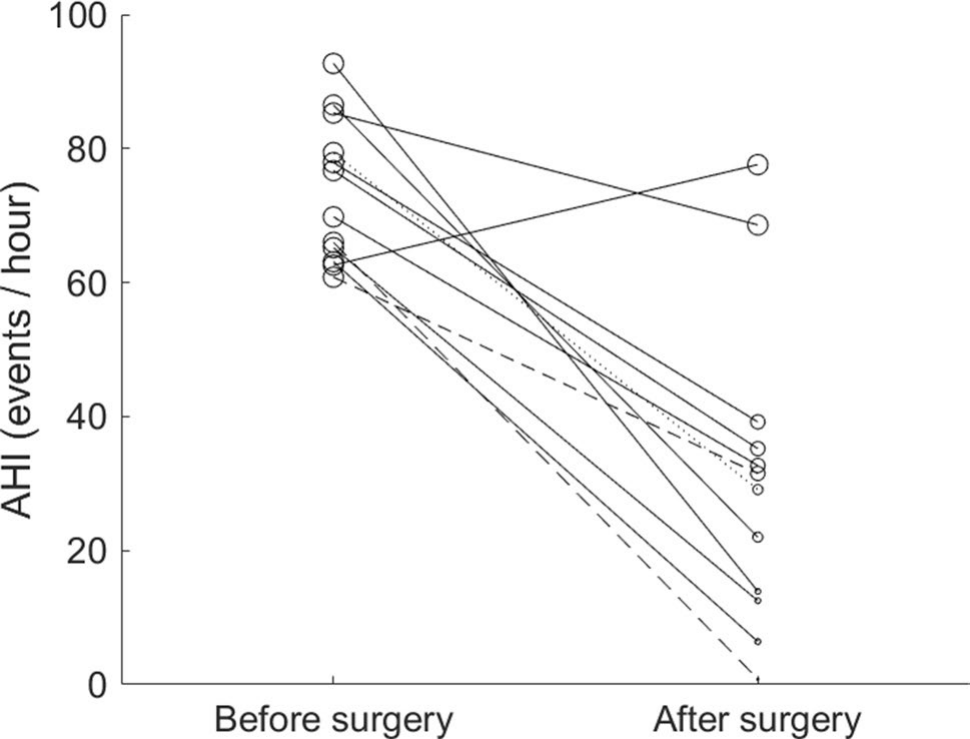
Individual change of apnea–hypopnea index (AHI) after the surgery. Dotted, dashed, and solid line: anatomical stage 1, 2, and 3, respectively. Very big, big, median, small, and none circle: in the category of very severe, severe, moderate, mild obstructive sleep apnea (OSA), and normal, respectively.

**Figure 4:**
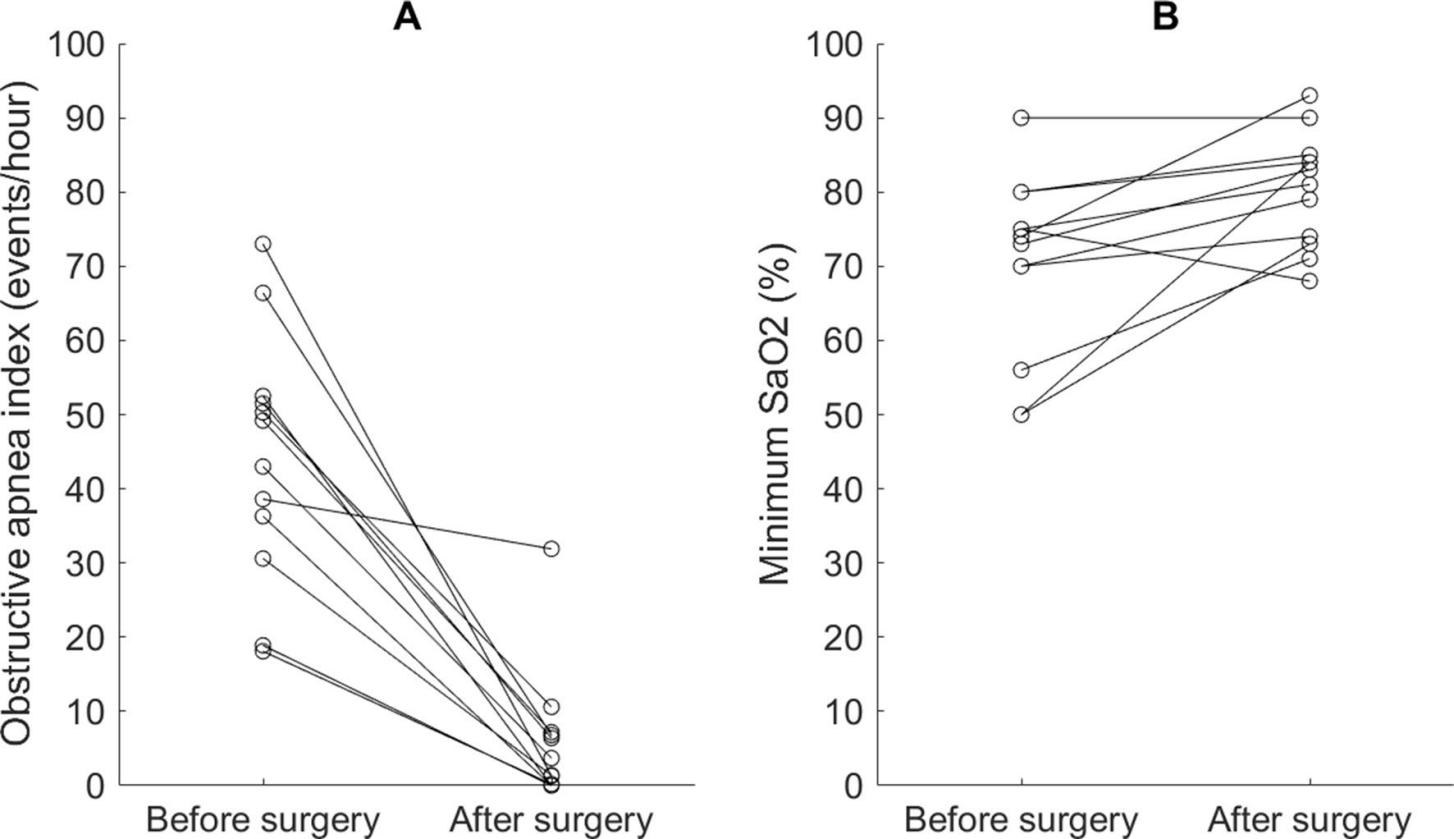
Individual change of obstructive apnea index (**A**) and minimum O2 saturation (**B**). *p* < 0.01 in both panels.

**Figure 5:**
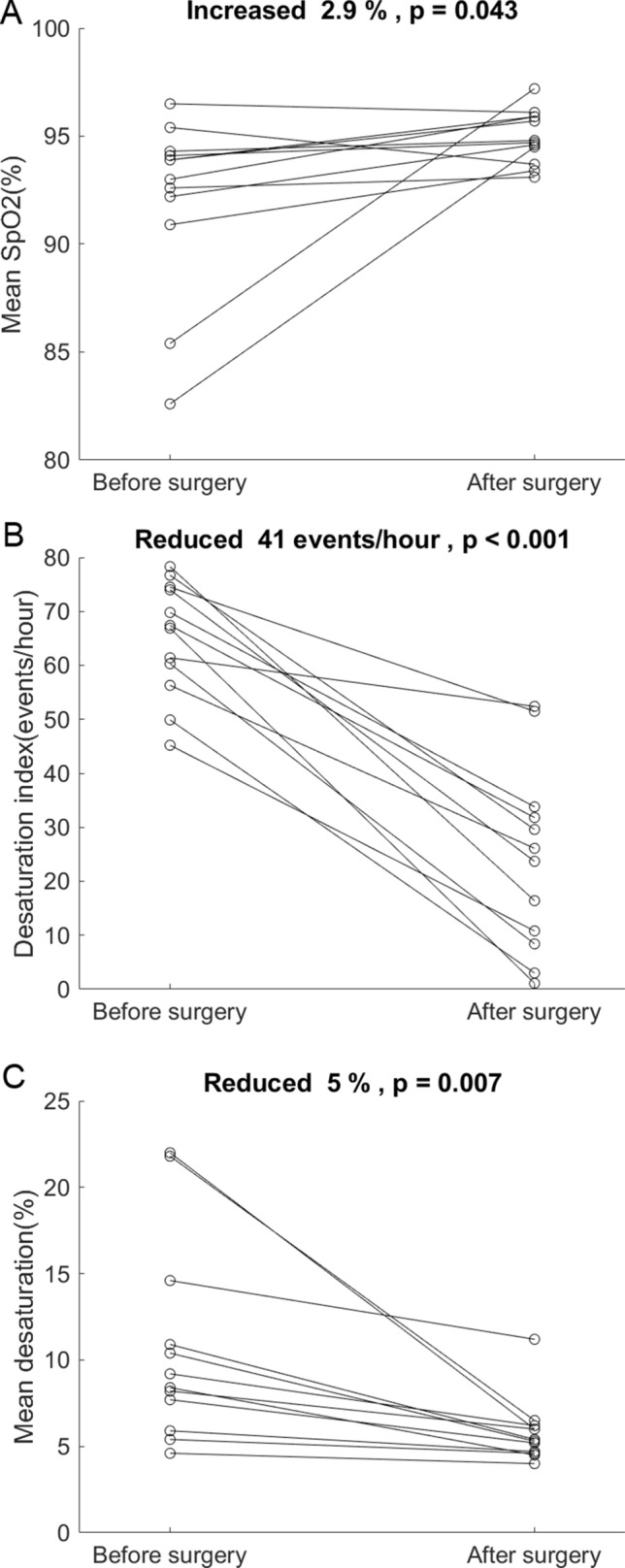
(**A**) Individual change of mean oxyhemoglobin saturation of pulse oximetry (SpO2) after the surgery. It reveals a ceiling effect. (**B**) Individual change of desaturation index after the surgery. The surgery reduced the mean desaturation index by 41 events/h (*p* < 0001). (**C**) Individual change of mean desaturation after the surgery.

The mean AHI (with one standard deviation) improved from 73.8 ± 10.7 events/h to 30.8 ± 23.2 after the surgery (Fig. 1). The AHI reduction was 58.3% and was away from 0 (*p* < 0.001). AHI reduction by Friedman anatomical stage was presented in Fig. 2. There were 1, 2, and 9 cases in stage 1, 2, and 3, respectively. Individual AHI change was shown in Fig. 3. One patient (8.3%) displayed normal PSG (AHI < 5 events/h) after the surgery. Three (25%) and 2 (16.7%) improved from very severe to mild and moderate categories, respectively. Four (33%) moved better to the severe level, and 2 (16.7%) remained in the very severe level. Ten out of 12 (83%) of the patients had various improvements on severity grouping. In the components of AHI, the surgery reduced OAI from 44.0 ± 16.8 to 5.8 ± 9.0 events/h, *p* < 0.001 (Fig. 4A) and improved minimum SpO2 from 70.3 ± 12.3 to 80.4 ± 7.7%, *p* = 0.0088 (Fig. 4B). It improved mean SpO2 from 92.1 ± 4.1 to 95.0 ± 1.2% (*p* = 0.0427), reduced desaturation index from 65.1 ± 10.7 to 24.1 ± 17.0 events/h (*p* < 0.001), and decreased mean desaturation from 10.8 ± 5.9 to 5.8 ± 1.9% (*p* = 0.007) (Fig. 5).

**Figure 6:**
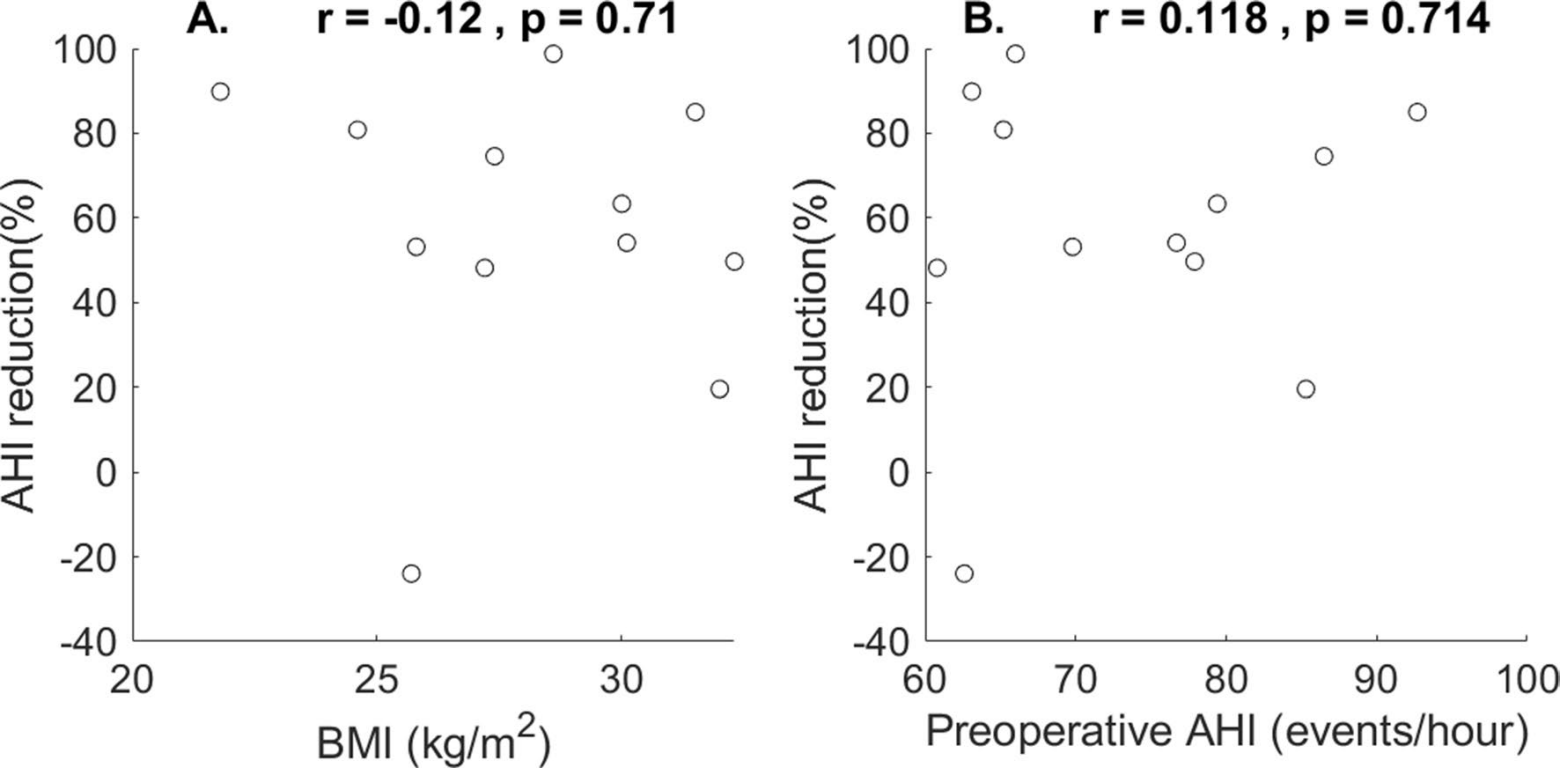
Scatter plot and correlation of preoperative body mass index (BMI) versus apnea–hypopnea-index (AHI) reduction (**A**) and preoperative AHI versus AHI reduction (**B**).

**Figure 7:**
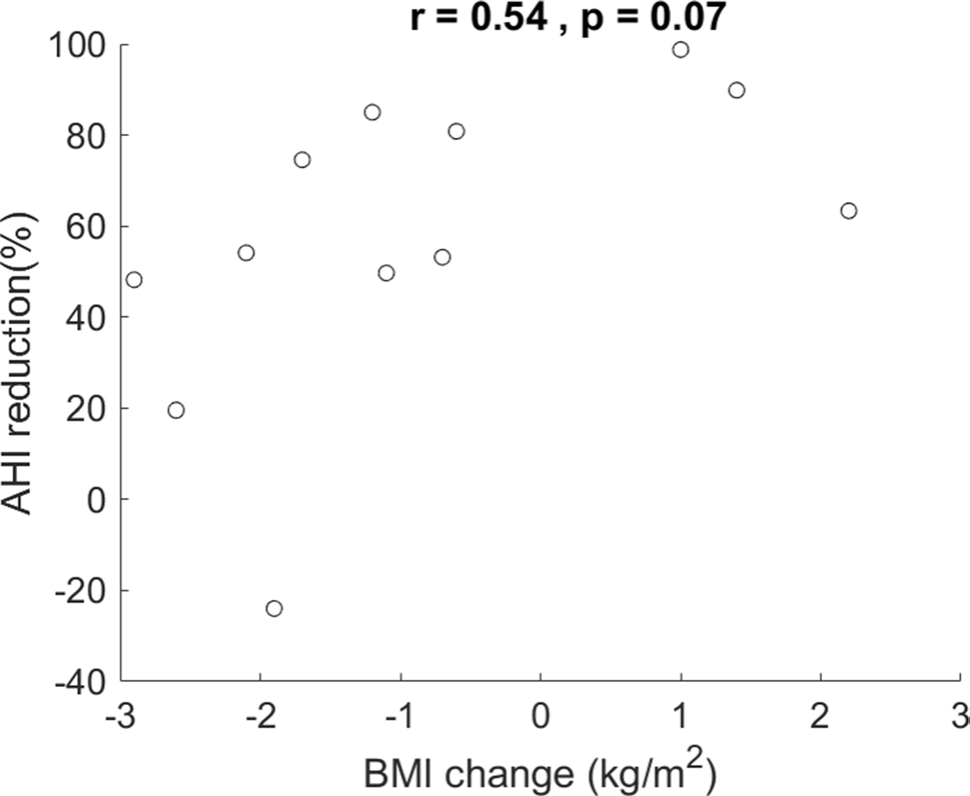
Scatterplot showed a trend of losing weight after the surgery but no statistic correlation between body-mass-index (BMI) change and apnea–hypopnea-index (AHI) reduction.

There was an event of left inferior tonsil wound hemorrhage in case 5 that resulted in an unplanned return to the operating room. No other major dehiscence or airway complication arose. The mean ESS score declined from 9.67 ± 5.23 to 7.83 ± 5.11. However, the difference did not reach statistical significance (*p* = 0.39 from a paired t-test). Scatter plots in Fig. 6 summarize the correlation tests of the two preoperative predictors. There was no statistical correlation between either of these two predictors and AHI reduction (r = − 0.12, *p* = 0.71 and r = 0.118, *p* = 0.714, respectively). Individual changes of BMI and AHI before and after the surgery were illustrated in Fig. 7. Although more patients (9 out of 12) lost weight after the surgery, BMI change was not statistically correlated with AHI reduction (r = 0.54, *p* = 0.07).

## Discussion

The results show that the multilevel surgery reduced AHI from 73.8 to 30.8 events/h, resulting in an AHI reduction of 58.3% (*p* < 0.001). It cut desaturation index from 65.1 to 24.1events/h (*p* < 0.001) and mean desaturation from 10.8 to 5.8% (*p* = 0.007). The surgery improved mean SpO2 from 92.1 to 95.0% (*p* = 0.0427) and minimum SpO2 from 70.3 to 80.4% (*p* = 0.0088). These results would help us on decision making with patients with very severe OSA with residual on-CPAP AHIs, or have a low level of daytime PaO2 but refuse the proposed direct skeletal surgery^13^, bariatric surgery^23^, or tracheostomy^24^, or refuse use of CPAP device.

A majority (83%) of the patients improved from the very severe category to milder ones and might reduce its comorbidity (e.g.,^30–35^). The rest (17% or 2 out of 12 patients) remained in the very severe group. The AHI made worse from 62.6 to 77.6 events/h after the surgery in case 7 and a little lessened from 85.4 to 68.6 events/h in case 6 (Table 1). To further understand the change made by the surgery in these 2 patients, we looked into matters of the PSGs before and after the surgery. In case 7 (the subject with worsened AHI after the surgery), the apnea part (i.e., OAI), improved from 38.6 to 31.9 events/h, and his minimum SpO2 improved from 50 to 84%. In case 6, the OAI reduced from 73 to 1.3 events/h, and his minimum SpO2 increased from 56 to 71%.

Minimum oxygen (O2) saturation has been listed as one of the main sleep-disordered breathing parameters besides AHI (e.g., see^17,29,37,39^) or used as one criterion to classify the severity of OSA (e.g., see^40–42^). So, we analyzed individual changes of minimum SpO2 in addition to OAI (Fig. 4). The mean OAI reduced from 44.03 to 5.79 events/h with a reduction rate of 86.8%, which showed that the OAI reduction (86.8%) was better than AHI reduction (58.3%). These results showed that the surgery reduced more portion of obstructive apnea than hypopnea. The sum of apneas and hypopneas per hour did not cut as many as the sum of apneas per hour—some obstructive apneas might become hypopneas after the surgery. The sample size of 12 in this study is small, although it is larger than 9 in Jacobowitz’s^43^, 6 in Walker’s^40^, 11 in Vilaseca’s^9^, and 10 in Mickelson’s^44^ for patients of very severe OSA. That limits generalizability of the results. It needs future studies with a larger sample size to verify the observation.

**Figure 8:**
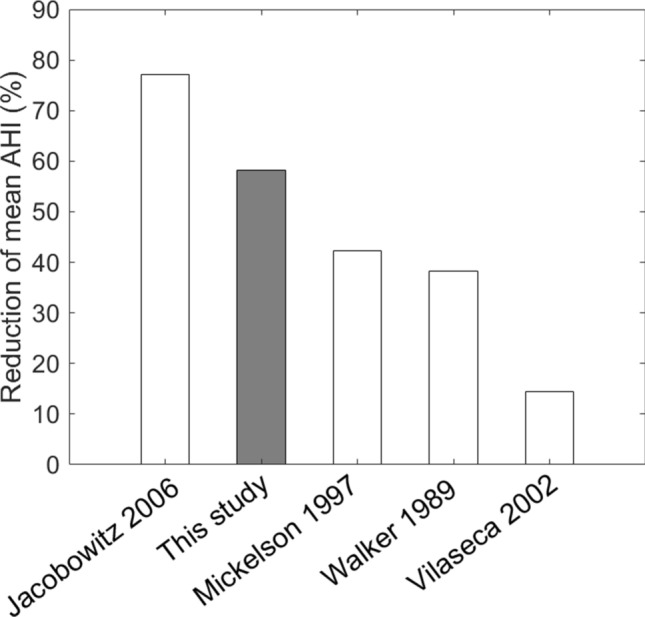
Comparison of surgical outcome in similar studies. See text for details.

To relate the surgical outcome with non-framework sleep surgeries in the literature, we reviewed 253 OSA related articles. Among them, 32 enrolled subjects with very severe OSA. Four provided detailed subject information and allowed us to calculate the mean AHI reduction. In Jacobowitz’s report^43^, mean AHI reduction was 77.1/h after UPPP with or without genioglossus advancement, hyoid suspension, or tongue-base radiofrequency treatment (n = 9), calculated from their Table 4. The mean AHI reduction was 38.2/h after UPPP (n = 6), computed from Table 1 in Walker’s study^40^; 14.4/h after UPPP and hyoid advancement with or without mandibular osteotomy with genioglossus advancement (n = 11), reckoned from Table 2 in Vilaseca report^9^. The mean RDI reduction was 42.2/h after UPPP and midline glossectomy with or without septomeatoplasty (n = 10), calculated from Table 1 in Mickelson’s study^44^. Figure 8 presents the comparison of the present study with these reports.

Classical surgical success—AHI < 20/h and AHI reduction > 50%, first proposed by Sher et al.^45^—was frequently reported in associated studies with less severity and so might serve as another outcome measure for comparison. The overall success rate in our study was 33% (4/12), which was lower than Jacobowitz’s 67% (6/9)^43^ but higher than Walker’s 17% (1/6)^40^, Vilaseca’s 9% (1/11)^9^, and Mickelson’s 30% (3/10)^44^.

Some studies recommend postoperative prudence treatment for patients with very severe OSA due to a higher risk of postoperative oxygen desaturation (e.g., Pang, K. P., Siow, J. & Tseng, P.^42^). They usually allocated these patients to the surgical intensive care unit (SICU) after the surgery (e.g., Rotenberg, B.^46^). In the present study, we cared for all patients in general ward areas with an oximeter monitor for 1 to 3 days while they breathe via the mouth because of nasal packing. No immediate or airway complication arose.

Some studies (e.g., Lin, H. S. et al.^36^) disclosed that BMI or preoperative AHI predicts surgical outcome. There was no correlation either between BMI and AHI reduction or between preoperative AHI and AHI reduction in this study. It needs future studies to find a good predictor for patients with very severe OSA.

## Conclusions

Our results show that modified Z-palatoplasty with one-layer closure in the one-stage multilevel surgery achieved a mean AHI and OAI reduction of 58.3% and 86.8% (*p* < 0.001), respectively. It reduced the frequency (desaturation index) and level (mean desaturation) of desaturation and improved mean and minimum oxygen saturation. Analyses show a shift in the components of breathing disturbances. This non-framework surgery reduced more apnea than hypopnea and might convert some apnea to hypopnea.

## Data Availability

The raw data in the current study are available from the supplementary information.
